# Multifaceted intestinal defense following experimental blunt abdominal trauma

**DOI:** 10.1007/s00068-026-03145-0

**Published:** 2026-03-17

**Authors:** Sophie Meisen, Farahnaz Rayatdoost, Katharina Oßwald, Annette Palmer, Markus Breunig, Markus Huber-Lang, Rebecca Halbgebauer

**Affiliations:** 1 Institute of Clinical and Experimental Trauma Immunology, Ulm University Medical Center, Helmholtzstr. 8/1, 89081 Ulm, Germany; 2 Department of Internal Medicine II, Ulm University, Ulm, Germany; 3 Institute of Molecular Oncology and Stem Cell Biology, Ulm University Hospital, Ulm, Germany

**Keywords:** Abdominal trauma, Intestinal injury, Intestinal barrier function, Immune response

## Abstract

**Purpose:**

Blunt abdominal trauma (AT) can cause significant intestinal injury and act as a trigger for remote posttraumatic organ dysfunction, including the development of multi-organ dysfunction syndrome (MODS). A deeper understanding of its impact on intestinal barrier integrity and immune regulation is essential for developing therapeutic strategies that support posttraumatic recovery.

**Methods:**

We assessed jejunal tissue 24 hours following a standardized murine blast-induced direct blunt AT. Histological, immunohistochemical, proteome profiling, Western Blot, and molecular analyses were applied to assess epithelial integrity, barrier-associated proteins, intestinal mucus composition, and immune responses.

**Results:**

The jejunum showed no trauma-specific histomorphological changes. Consistently, the expression of key epithelial barrier proteins, such as E-cadherin and tight-junction protein 1, remained stable or were even upregulated. Local inflammatory responses were evident; AT induced activation of defense-related pathways, accompanied by a marked increase in S100A8 expression and enhanced infiltration of CD3^+^ T cells. Mucus-covered areas of the intestinal surface were reduced, although transcriptional levels of MUC2 and C1GALT1 were elevated. In addition, the antimicrobial peptide CRAMP was upregulated in jejunal tissue following injury.

**Conclusions:**

These findings suggest that the intestine initiates a multifaceted protective response to blunt AT, characterized by barrier preservation, mucus alterations, and adaptive immune activation. Remarkably, although the trauma was sufficient to elicit an inflammatory reaction, it simultaneously activated mechanisms that reinforced epithelial integrity and compensatory processes that may counteract barrier disruption in the early posttraumatic phase.

**Supplementary information:**

The online version contains supplementary material available at 10.1007/s00068-026-03145-0.

## Introduction

Trauma remains a leading global health concern, accounting for one in ten deaths across all age groups and sexes, and is the primary cause of mortality in individuals under the age of 35 [1]. Although abdominal trauma (AT) is less frequent among trauma-related injuries, reported in approximately 6% to 41% of severely injured patients [2–4], it can cause severe consequences for intestinal function and may trigger remote multi-organ complications [5]. Blunt AT, predominantly resulting from traffic accidents, accounts for approximately 90% of abdominal injuries [2, 6, 7], surpassing penetrating trauma from stab wounds or firearms [8]. In pediatric patients, blunt AT is often linked to transport incidents and falls, and, to a lesser extent, to child abuse [6]. Clinical diagnosis of blunt AT can be challenging due to its subtle presentation, which may lead to delayed detection and increased morbidity and mortality beyond the acute post-injury phase [9, 10].

The intestine, in danger by the inflicted trauma force vector of blunt AT, functions as a selective barrier against bacterial intrusion while orchestrating nutrient absorption and metabolic waste excretion [11]. This barrier comprises intestinal epithelial cells (IECs), goblet cell-derived mucus, antimicrobial peptides (AMPs) and mucosa-associated lymphatic tissue. Tight and adherens junctions between IECs regulate paracellular transport and maintain barrier integrity. Together, these elements create a physical and chemical shield, safeguarding sterile host tissues from pathogenic bacteria [12–14]. The jejunum, the central segment of the small intestine, is characterized by its highly folded mucosa and villi, which optimize surface area for digestion and absorption, thereby positioning it as a pivotal site for nutrient absorption [15]. Due to its large surface area and rich vascularization, the jejunum is particularly well suited for investigating intestinal barrier dysfunction. Moreover, jejunal villi are highly susceptible to hypoperfusion, making this region especially relevant in the context of trauma [16]. Microbial colonization is also considerably lower in the jejunum than in the colon, thereby reducing the confounding influence of microbial interactions on experimental outcomes [17]. Despite its physiological importance, the jejunum has received limited attention in trauma research, particularly in the context of direct blunt AT.

Disruption of the intestinal barrier following trauma can lead to bacterial translocation, triggering immune activation and a pro-inflammatory cascade that exacerbates intestinal injury. This may further escalate into systemic inflammation, contributing to remote organ dysfunction and increasing the risk of MODS [18, 19]. Therefore, we aimed to investigate early trauma-induced alterations of intestinal barrier integrity and immune pathophysiology in a previously established murine model of blunt abdominal injury [20, 21], with a focus on direct local intestinal processes rather than systemic or remote organ alterations. In previous studies, AT was applied at different intensities and macroscopic injury of intra-abdominal organs was observed across all trauma intensities. Furthermore, posttraumatic lethality rates ranged up to 18% at the highest trauma intensity and to 9% at the intermediate intensity [20], the latter corresponding to the trauma intensity examined in the present study. Moreover, AT application induced an early increase in systemic IL-6 [20] and I-FABP [21], suggesting systemic inflammation as well as intestinal barrier breakdown. Based on these findings, we hypothesized that direct AT causes a subacute posttraumatic impairment of the intestinal barrier function in the jejunum alongside an activation of intestinal inflammation.

## Methods

### Animals

In line with the 3 R-principles, we performed a post-hoc analysis of jejunal tissue samples obtained from a previously published study [20]. All procedures were conducted in accordance with the current Animal Welfare Experimental Animal Ordinance with the approval of the Tübingen Regional Council (TVA Reg. No. 1345). Blunt AT was applied to male C57BL/6JRj mice (Janvier Labs, Le Genest-Saint-Isle, France). Eight- to 12-week-old mice weighing 20–30 g were selected either as experimental or sham animals (*n* = 5). Animals had free access to water, food, and nesting material, and were kept under a 12/12 h light-dark cycle.

### Anesthesia and blunt AT

Opioid analgesia was provided with subcutaneous buprenorphine (Temgesic®, Boehringer, Mannheim, Germany) at a dose of 0.05 mg/kg body weight, administered 30 min prior to trauma induction or sham treatment. Throughout the ensuing observation period, supplementary doses were administered at 8, 16, and 20 h post-intervention. Anesthesia was induced with 4 vol% sevoflurane (Abbott, Wiesbaden, Germany) in 96 vol% oxygen and maintained via a nasal mask. The experimental apparatus for blunt trauma induction consisted of a two-part cylinder divided by a thin polyester membrane (DuPont, Wilmington, USA). A distance of 2.6 cm was set between the cylinder and the mice abdomen. The upper chamber, connected to a compressed air cylinder, was pressurized until the membrane ruptured at 0.83 ± 0.27 bar, releasing a targeted blast of compressed air that caused abdominal compression without external skin injuries. To prevent post-traumatic hypothermia, animals were placed in a heated cage during recovery from anesthesia. In addition to the experimental animals, sham animals underwent an identical experimental procedure except for the application of the AT. Accordingly, sham mice received buprenorphine injections and brief sevoflurane anesthesia, and tissues were collected 24 h afterwards.

### Harvest and tissue preparation

Organ harvesting took place 24 h post-trauma or sham procedure after induction of deep sevoflurane anesthesia and exsanguination by cardiac puncture. The abdomen was opened for organ retrieval and after ligation, the jejunum was separated from the other intestinal sections. An approximately 3 cm long segment of the jejunum was fixed in 3.7% formaldehyde solution for 24 h, then rinsed, dehydrated through a graded alcohol series, and embedded in paraffin. Organ sections of 4 µm thickness were cut using a microtome for histological analysis. To preserve additional intestinal tissue, the lumen of the jejunum was rinsed thoroughly with cold PBS and stored at −80 °C. Bronchoalveolar lavage fluid (BALF) was collected by flushing the left lung with 0.5 mL sterile phosphatebuffered saline (PBS) and centrifugation for 10 min at 400 g and 4 °C, and then stored at −80 °C for further analysis. EDTA blood samples were centrifuged for 5 min at 800 g and 4 °C and 2 min at 13,000 g and 4 °C. Plasma was collected and stored at −80 °C.

### Hematoxylin and eosin (H&E) and periodic acid-schiff (PAS)-Alcian blue staining

Jejunal sections were deparaffinized in xylene and rehydrated through a graded ethanol series. For H&E staining, sections were placed in hematoxylin solution modified according to Gill III° (Sigma Life Sciences, St. Louis, USA) for 6 min, rinsed in water and 0.5% hydrochloric acid, and stained with 1% eosin solution (Morphisto, Frankfurt am Main, Germany) for 30 s. For PAS-Alcian blue staining, tissue sections were stained with Alcian blue solution (Sigma Life Sciences, St. Louis, USA) for 5 min, followed by periodic acid solution for 10 min, Schiff’s reagent for 15 min and hematoxylin solution modified according to Gill III° for 20 s. Rinsing was performed between all step. Finally, H&E- and PAS-Alcian blue-stained sections were dehydrated and mounted using Neo-Mount™ mounting medium (Sigma Life Sciences, St. Louis, USA).

### Immunohistochemical staining

For immunohistochemical staining, jejunal sections were deparaffinized and rehydrated, followed by antigen retrieval by boiling in either citrate buffer (pH 6.0; for MPO staining) or EDTA buffer (pH 9.0; for CDH1 and CD3 staining) for 7 min. After cooling, sections were rinsed in distilled water and Tris-buffered Saline (TBS), and subsequently permeabilized with 0.2% Triton X-100 (Sigma-Aldrich, St. Louis, USA) for 10 min. To minimize autofluorescence, tissue sections were treated with Sudan Black B (Sigma-Aldrich, St. Louis, USA) for 20 min, followed by rinsing in TBS and TBST (TBS with 1% Tween-20). Non-specific binding sites were blocked using antigen block solution (see Suppl. Table 1). Primary antibodies were applied overnight at 4 °C at the following dilutions: 1:200 for MPO (Human/Mouse Myeloperoxidase/MPO Antibody, R&D Systems, Minneapolis, USA), 1:100 for CDH1 (E-cadherin [24E10] Rabbit IgG monoclonal antibody, Cell Signaling Technology, Boston, USA), and 1:250 for CD3 (Anti-CD3 antibody [CD3-12] Rat monoclonal antibody, Abcam, Cambridge, UK). After washing, sections were incubated with the corresponding secondary antibody for 60 min. For MPO, Donkey anti-Goat IgG (H+L), Alexa Fluor™ 568 (1:200); for CDH1, Alexa Fluor™ 488-conjugated Goat anti-Rabbit IgG (1:300); and for CD3, Goat anti-Rat IgG conjugated to Alexa Fluor™ 568 (1:300) were used (all antibodies from Thermo Scientific, Rockford, USA). The tissue sections were covered with Prolong Gold Antifade with DAPI (Life Technologies Corporation, Eugene, USA) and dried overnight at 4 °C. Imaging was performed using a Zeiss Axio Imager M1 light microscope with AxioVision SE64 Rel. 4.9 software (Zeiss, Oberkochen, Germany).

### Histological analysis

QuPath-0.4.4 software [22] was used for quantification of mucus-covered areas and goblet cells. Images of PAS-Alcian blue-stained jejunal tissue sections were taken at 200x magnification. For subsequent autonomous area recognition by the software, six randomly selected images were used as training data by manually labeling mucus-covered areas. For each animal, ten villi were selected and included in the analysis.

### Quantitative polymerase chain reaction (qPCR)

Jejunal tissue was gently pulverized using an RNase-decontaminated (RNaseZAP™, Sigma-Aldrich, St. Louis, USA) mortar and pestle on liquid nitrogen to prevent autolysis and kept on ice until RNA extraction. Total RNA was isolated using the RNeasy Plus Kit (Qiagen, Hilden, Germany) according to the manufacturer’s instructions. RNA concentration was determined using the Qubit™ RNA High Sensitivity Assay Kit (Qiagen, Hilden, Germany), following the manufacturer’s protocol.

Complementary DNA (cDNA) was synthesized from 500 ng of RNA per sample using the AffinityScript qPCR cDNA Synthesis Kit (Agilent, Santa Clara, USA) in accordance with the manufacturer’s instructions. The cDNA was diluted with RNase-free water and stored at −20 °C. To determine alterations in gene expression between sham and experimental group, qPCR analyses were performed in duplicate. The procedure was conducted according to the manufacturer’s instructions using the Brilliant III SYBR-Green qPCR Master Mix Kit (Agilent, Santa Clara, USA) and AriaMx Real-time PCR-System (Agilent, Santa Clara, USA). For evaluation of the qPCR results, the AriaMx HRM qPCR software (Agilent, Santa Clara, USA) was used. Primers used for gene expression analysis are listed in Suppl. Table 1. Relative fold-change expression levels were calculated using the 2^-ΔΔCT^ method, with *Actb* serving as the reference gene.

### Tissue homogenization and total protein concentration

Frozen jejunal tissue samples were lysed in 200 µl RIPA buffer using innuSPEED Lysis Tubes P (Innuscreen GmbH, Berlin, Germany) and homogenized with the SpeedMill Plus tissue homogenizer (Analytik Jena, Jena, Germany). Homogenates were shock-frozen, before they were thawed and centrifuged at 16,000 g for 15 min at 4 °C. The resulting supernatant was kept on ice for the subsequent steps. Total protein concentration was determined using the PIERCE BCA Protein Assay Kit (Thermo Scientific, Rockford, USA) according to the manufacturer’s instructions.

### Enzyme-linked immunosorbent assay (ELISA)

To measure the concentrations of C5a in plasma and BALF, a sandwich ELISA was performed using the C5a ELISA Kit Mouse Duo Set (R&D Systems, Minneapolis, USA). For the BALF samples, the BCA Protein Assay Kit (#23225; Thermo Fisher Scientific, Inc.) was used to determine the total amount of protein. The experiments were performed according to the manufacturer’s instructions.

### Proteome profiler

Cytokine expression on a protein level was determined semi-quantitatively using the Proteome Profiler Mouse XL Cytokine Array Kit (R&D Systems, Minneapolis, USA). Membranes were imaged using the ChemiDoc™ XRS+ System (Bio-Rad Laboratories, Hercules, USA), and analyzed with ImageLab software (Bio-Rad Laboratories, Hercules, USA). Pathway analysis of differentially regulated cytokines was performed using ShinyGO v0.82 (https://bioinformatics.sdstate.edu/go/).

### Western blotting

Tissue homogenates prepared in RIPA buffer were diluted 1:2 with 2x Laemmli Sample Buffer, before incubation for 5 min at 95 °C and electrophoresis using Mini-PROTEAN® TGX™ Stain-Free Gels (4–20%) (Bio-Rad Laboratories, Hercules, USA). Proteins were transferred to Hybond P-PVDF membranes using the Trans-Blot Turbo Transfer System (Bio-Rad Laboratories, Hercules, USA). To block non-specific antibody binding sites, membranes were incubated in 5% milk for 1.5 h, followed by overnight incubation at 4 °C with a 1:1500 dilution of CAMP polyclonal antibody (Proteintech, Manchester, UK) in 5% milk or a 1:500 dilution of Cleaved Caspase-3 (Asp175) Antibody (Cell Signaling Technology, Boston, USA) in 5% milk, respectively. Membranes were washed three times with TBST, incubated with StrepTactin-HRP-conjugated secondary antibody (Bio-Rad Laboratories, Hercules, USA) for 60 min and washed again in TBST and TBS. Clarity Western ECL Substrate (Bio-Rad Laboratories, Hercules, USA) was applied, and membranes were imaged using the ChemiDoc™ XRS+ System (Bio-Rad Laboratories, Hercules, USA). ReBlot Plus Strong Antibody Stripping Solution (Merck KGaA, Darmstadt, Germany) was applied for 15 min, membranes were blocked with 5% BSA for 30 min and incubated overnight at 4 °C with β-Actin antibody (Cell Signaling Technology, Boston, USA) in a 1:1500 dilution with 5% BSA. Following rinsing with TBST, membranes were incubated with StrepTactin-HRP-conjugated secondary antibody for 60 min, rinsed again and Clarity Western ECL Substrate was applied for subsequent imaging.

### Statistical analysis

Data were analyzed using GraphPad Prism version 10.0.0 for Windows (GraphPad Software, Boston, USA). Results are presented as individual values and mean ± standard error of the mean (SEM). Due to low animal numbers, a normal distribution was not assumed. Group differences were assessed using the Mann-Whitney U test for non-parametric comparison between two groups. Two-sided *p*-values < 0.05 were considered statistically significant.

## Results

### Absence of trauma-specific histological damage in the jejunum 24 h after blunt AT

**Fig. 1:**
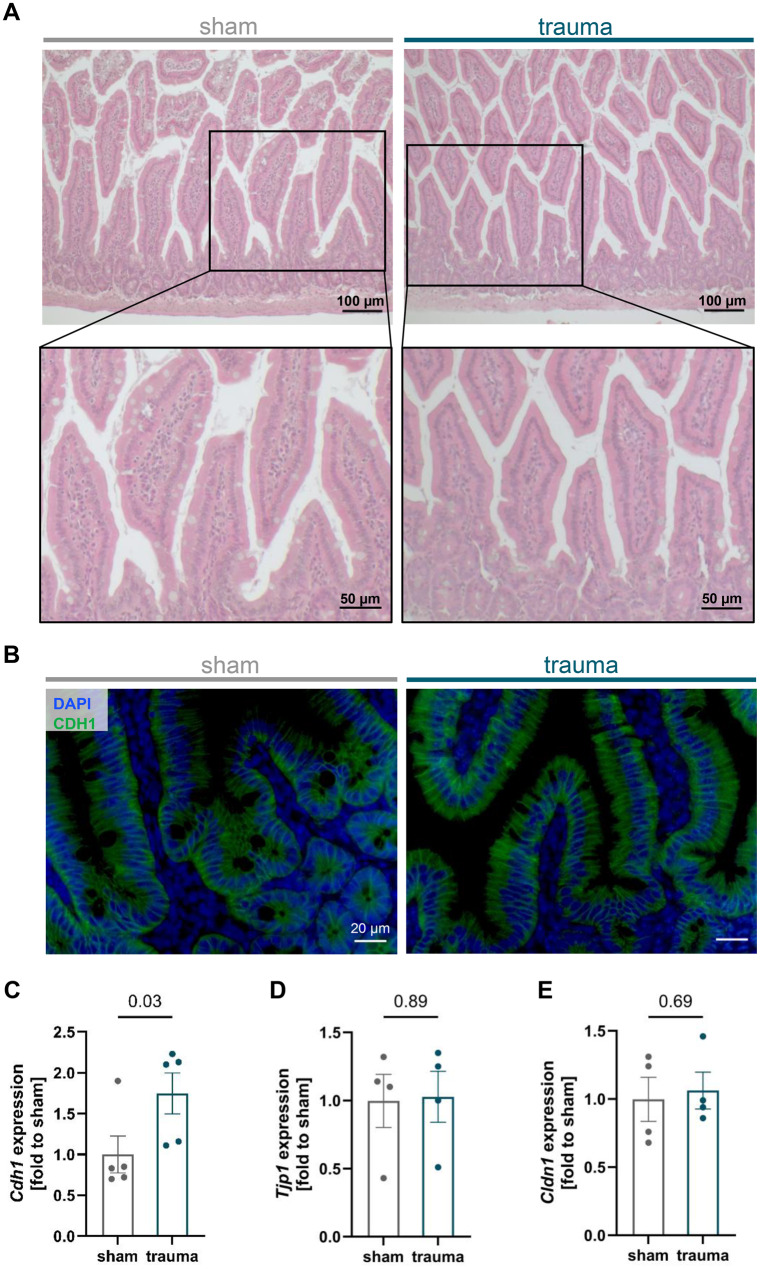
Histological and molecular assessment of epithelial integrity and barrier protein expression in the murine jejunum following blunt abdominal trauma. (**A**) Jejunal tissue sections stained with hematoxylin and eosin (H&E) 24 h after trauma or sham intervention showed no distinct morphological alterations indicative of trauma-induced epithelial damage. (**B**) Immunohistochemical analysis of E-cadherin (CDH1) revealed comparable overall protein expression between groups. (**C-E**) Gene expression profiling demonstrated a significant upregulation of *Cdh1* in traumatized mice, while tight junction protein 1 *(Tjp1/Zo-1)* and claudin-1 *(Cldn1) *remained unchanged. *n* = 4–5 per group. Statistical analysis was performed using the Mann-Whitney U test, with significance defined as *p* < 0.05. Data are presented as mean ± standard error of the mean (SEM)

To assess potential tissue injury following blast-induced blunt AT, histological analysis was performed on H&E-stained jejunal sections from sham and trauma mice 24 h post-intervention (Fig. 1A). This initial evaluation aimed to determine whether blunt AT induces structural damage to the intestinal epithelium and villus architecture. Microscopic examination revealed no histological differences between the two groups and did not provide conclusive evidence of trauma-specific damage to the epithelial integrity, for instance lifting of the epithelial layer from the lamina propria, epithelial defects reaching beyond lamina muscularis mucosae, or crypt loss. Furthermore, gene expression of the apoptosis and necroptosis regulators thirty-eight negative kinase 1 *(Tnk1)* and receptor-interacting serine/threonine-protein kinase 3 *(Ripk3),* respectively, remained unchanged compared to sham controls (Suppl. Fig. 1 A, B). Consistently, western blot analysis demonstrated unaltered levels of cleaved caspase‑3 in jejunal tissue (Suppl. Fig. 1 C, D), indicating no significant induction of tissue apoptosis.

### Post-traumatic regulation of epithelial junction proteins in the jejunum

Next, we assessed the post-traumatic expression of junction proteins, which constitute essential components of the intestinal barrier and serve as modulators of paracellular transport. Protein quantification showed no significant change in E-cadherin (CDH1) protein expression after trauma (Fig. 1B). Interestingly, transcriptional analysis uncovered a significant upregulation of *Cdh1* gene expression (Fig. 1C). In contrast, the gene expression levels of the tight junction proteins tight junction protein 1/zonula occludens (*Tjp1/Zo-1*) and claudin-1 (*Cldn1*) were not modulated after traumatic exposure (Fig. 1D, E). Consistent with these observations, no evidence of bacterial translocation was found in the splenic tissue of injured mice at 24 h post-trauma as assessed by 16S rRNA qPCR (Suppl. Fig. 2).

### Involvement of inflammatory pathways in jejunal tissue of traumatized mice

**Fig. 2:**
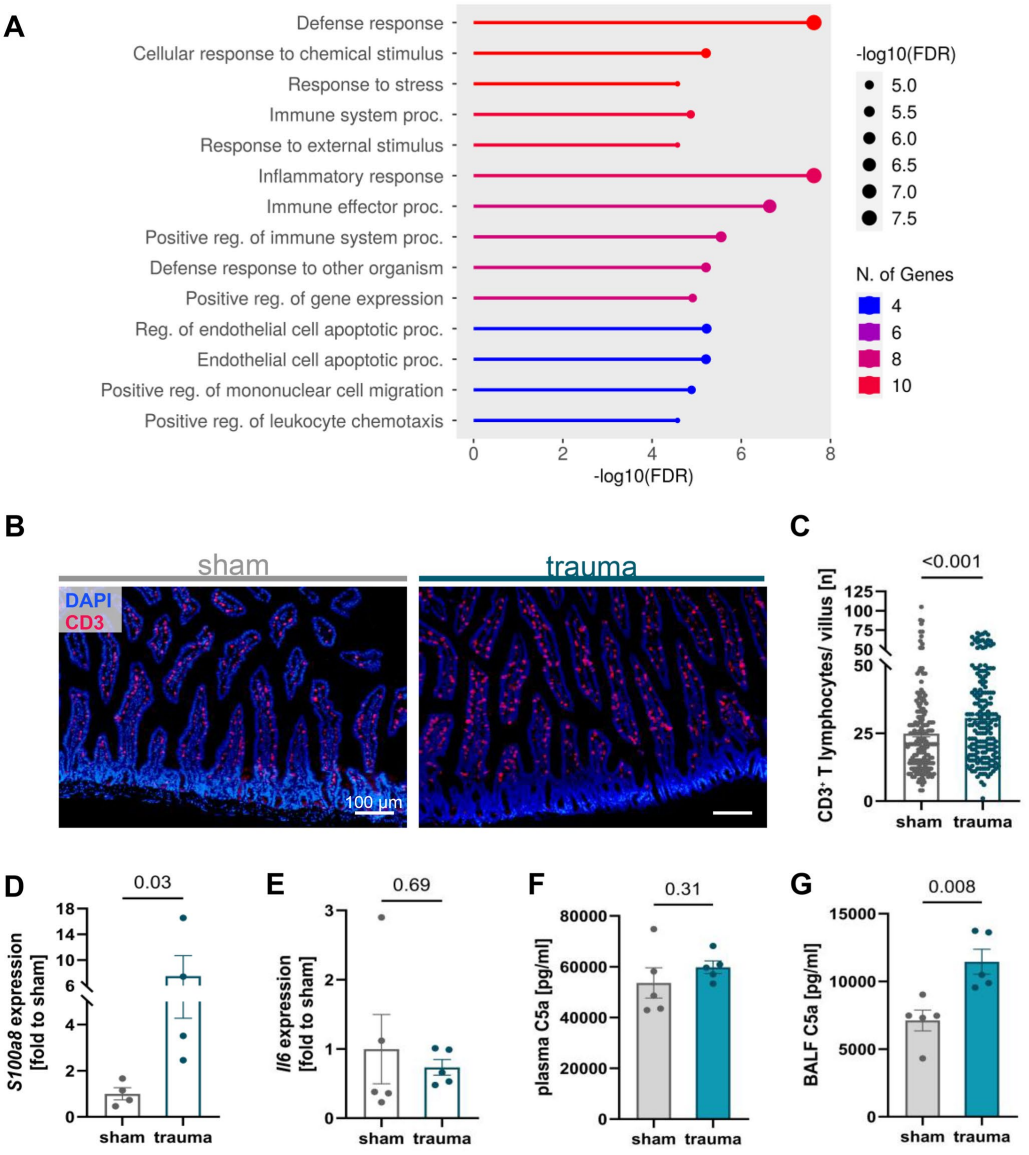
Posttraumatic defensive pathway activation and cellular immune response in the murine jejunum. (**A**) Proteome profiler-based pathway analysis revealed activation of immune and defense-related mechanisms. The 15 pathways with the highest degree of regulation are shown. (**B, C**) Histological quantification in jejunal tissue demonstrated a significant increase of CD3^+^ T lymphocytes in the trauma group relative to controls. (**D**) Gene expression profiling of *S100a8* showed a significant post-traumatic upregulation in traumatized mice compared to sham animals. (**E**) In contrast, no statistically significant alteration was observed in interleukin-6* (Il6)* expression. (**F**) Plasma levels of C5a were unaltered by traumatic exposure. (**G**) C5a in bronchoalveolar lavage fluid (BALF) was significantly increased in mice 24 h after AT compared to shams. **C**: *n* = 180 per group; **D-G**: *n* = 4–5 per group. Statistical analysis was performed using the Mann-Whitney U test, with significance defined as *p* < 0.05. Data are presented as mean ± standard error of the mean (SEM)

Proteome profiling combined with pathway analysis of jejunal tissue 24 h following blunt AT revealed an enrichment of defense-related and inflammatory pathways, as well as endothelial damage (Fig. 2A). Blunt AT led to changes of the adaptive immune response as evidenced by an increased count of CD3^+^ lymphocytes 24 h after trauma (Fig. 2B, C). Subsequent qPCR analysis indicated a marked upregulation of *S100a8* gene expression in traumatized mice compared to sham-treated animals (Fig. 2D) while immunohistochemical staining for MPO indicated unchanged neutrophil counts (Suppl. Fig. 3A, B). Concomitant with these findings, there was no significant increase in gene expression levels of C-X-C motif chemokine ligand 1 (*Cxcl1*), a key neutrophil chemoattractant, between trauma and sham groups (Suppl. Fig. 3C). No significant alteration was observed in interleukin-6 (*Il6*) gene expression following trauma in comparison to sham-treated animals (Fig. 2E). Levels of C5a in plasma were unaltered by AT. In contrast, local inflammation of the lung, assessed by C5a level measurements in BALF, was increased post-trauma (Fig. 2F, G).

### Posttraumatic changes of mucus expression

**Fig. 3:**
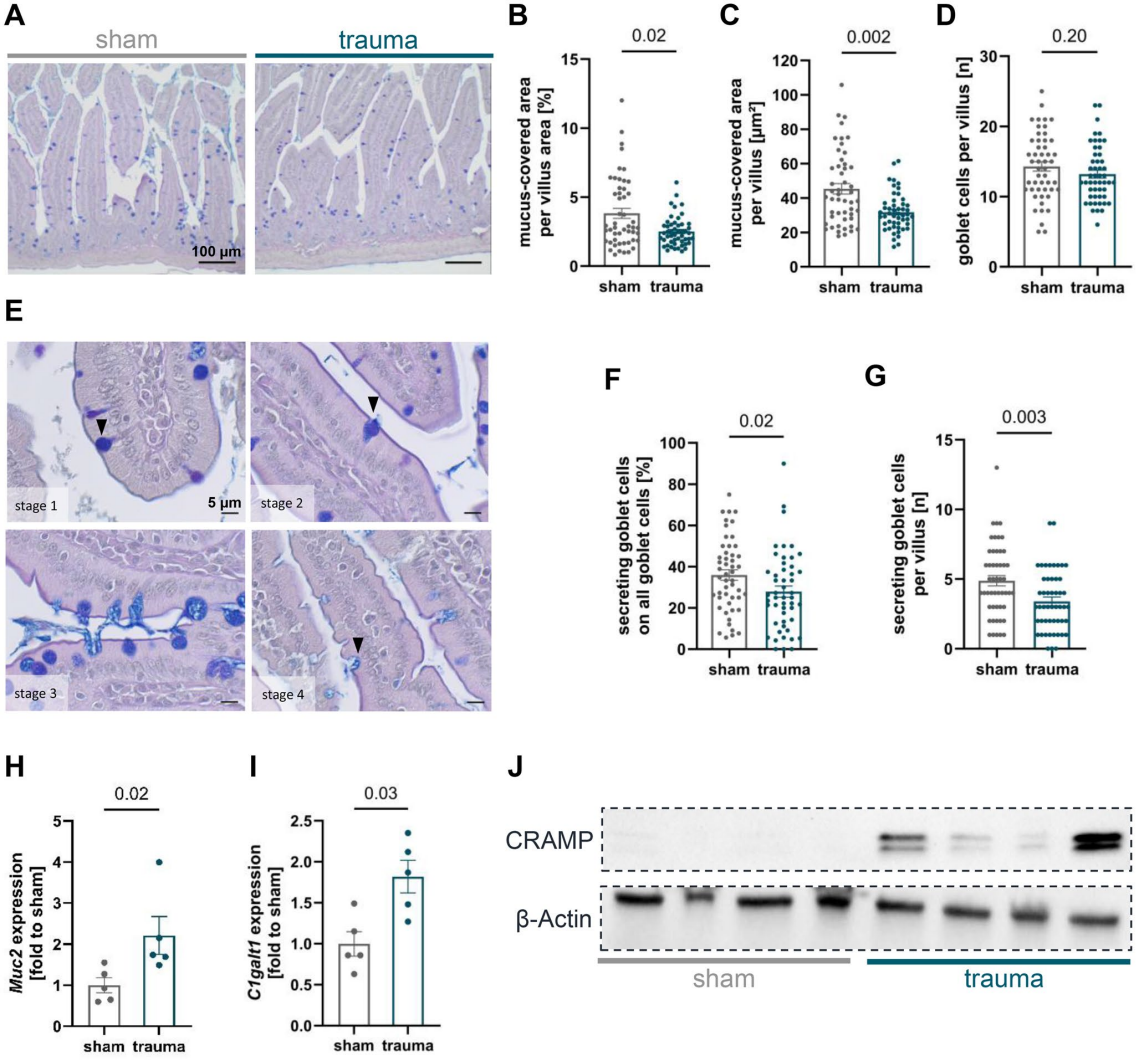
Alterations in jejunal mucus and antimicrobial peptide expression following trauma. **(A**) Representative PAS-Alcian blue-stained sections were used to quantify intestinal mucus content in the jejunal tissue. (**B, C**) Trauma resulted in a significant reduction in the mucus-covered area compared to sham-treated mice. (**D**) The average number of goblet cells per villus did not differ significantly between control and trauma-exposed mice. (**E**) Goblet cell secretory stages are illustrated, ranging from mucus-filled cells to initiation of secretion, active secretion, and post-secretion emptied cells. (**F, G**) Trauma-exposed mice showed a significantly lower proportion of secretory goblet cells relative to the total goblet cell population, as well as an absolute reduction in actively secreting goblet cells. (**H, I**) Furthermore, trauma resulted in a significant upregulation of mucin 2 (*Muc2*) and core 1 β1,3-galactosyltransferase 1 (*C1galt1*) expression compared to sham animals. (**J**) Western blot analysis of Cathelicidin-related antimicrobial peptide (CRAMP; ~18 kDa) in jejunal tissue revealed a marked increase following trauma compared to sham-treated mice. **B-D, F, G**: *n* = 50 (10 villi per animal); **H-J**: *n* = 4–5. Statistical analysis was performed using the Mann-Whitney U test, with significance defined as *p* < 0.05. Data are presented as mean ± standard error of the mean (SEM)

Goblet cell-derived mucus contributes critically to intestinal barrier integrity by forming both physical and chemical protective layers along the epithelial surface. Therefore, we assessed the impact of blunt AT on mucus expression as visualized by PAS-Alcian blue staining (Fig. 3A). Following traumatic injury, mice exhibited a significant reduction in mucus-covered area relative to the villus area compared to sham animals (Fig. 3B), reflecting a diminished goblet cell filling. Moreover, a significant decrease in the absolute mucus-covered area was observed 24 h post-trauma when compared with sham counterparts (Fig. 3C). To rule out that the mucus area reduction was attributable to a diminished goblet cell population, goblet cells were counted. However, goblet cell numbers remained stable following trauma in comparison to the sham group (Fig. 3D). Furthermore, goblet cells were classified according to their secretory stage, ranging from cells with filled mucus reservoirs to actively secreting cells and those exhibiting a depleted morphology (Fig. 3E). Mice subjected to trauma exhibited a decreased proportion of goblet cells in secretory states relative to the total goblet cell population, alongside a significant reduction in the absolute number of secreting goblet cells, compared to sham mice (Fig. 3F, G). Following blunt abdominal injury, mice exhibited a significant upregulation of mucus-associated genes including mucin 2 (*Muc2*) and core 1 β1,3-galactosyltransferase (*C1galt1*) in comparison to sham mice (Fig. 3H, I). Protein levels of Cathelicidin-related antimicrobial peptide (CRAMP), the murine homolog of human CAMP/LL-37, were also elevated post-trauma (Fig. 3J). The complete membranes are shown in Suppl. Fig. 4.

## Discussion

In our murine model of direct blunt AT, we focused on the intestine, a site where the luminal microbiome lies in close anatomical proximity and strict delineation to sterile host tissue, highlighting its potential role as a critical mediator in the post-traumatic onset of MODS [5]. Therefore, we aimed to characterize posttraumatic alterations in intestinal barrier function of the jejunum and to elucidate the local defense and immune response within 24 h after blunt AT. Initial analyses focused on epithelial barrier proteins to assess potential micro-structural compromise. Posttraumatic intestinal barrier dysfunction can occur within hours and may persist for several days depending on the trauma type and severity. Previous studies have demonstrated elevated plasma claudin-5 levels at 6 h and 24 h after blunt AT [21] as well as intestinal leakage early following murine extra-abdominal polytrauma and hemorrhagic shock [23]. Plasma levels of tight junction proteins and I‑FABP remain elevated for several days in critically ill patients, indicating prolonged intestinal barrier dysfunction [24, 25]. Likewise, marked intestinal injury with epithelial apoptosis and shedding has been described after traumatic brain injury (TBI), accompanied by elevated serum endotoxin levels on the third post‑traumatic day [26]. Based on these findings, we anticipated detectable barrier alterations 24 h after blunt AT in our model. However, one study reported complete restoration of intestinal barrier integrity within 24 h after hemorrhagic shock [27], highlighting the remarkable capacity for rapid intestinal epithelial restitution. In the present model, we did not observe a reduction in key adherens or tight junction proteins, suggesting that trauma did not compromise the micro-architecture of the epithelial barrier at this time point. Furthermore, no increase of epithelial apoptosis markers such as cleaved caspase-3 or *Tnk1* was observed. Although post-traumatic loss of intestinal tight junction proteins is frequently observed [28–30], it appears to be dependent on direction and magnitude of the applied traumatic force. For instance, post-traumatic loss of ZO-1 has been observed following polytrauma and TBI [23, 30]. Interestingly, both ZO-1 expression and intestinal barrier function were fully restored within 2 h following ischemia-reperfusion injury [31]. Such preservation and rapid restoration of junctional proteins may result from a complex interplay of compensatory mechanisms that stabilize barrier integrity after injury. In our study, E-cadherin showed unaltered protein levels following blunt AT, whereas gene expression was significantly elevated. This discrepancy may reflect dynamic regulation at post-transcriptional levels [32]. A similar transient change in CDH1 expression has been observed following surgical trauma, with increased expression noted 24 h post-injury [33]. In line with these results, we found no evidence of bacterial translocation into systemic compartments as demonstrated by the absence of bacterial 16S rRNA in splenic tissue samples. This is in accordance with our previous study in which AT – even when applied with a higher intensity – did not lead to significantly increased histological damage. Notably, barrier injury as measured by elevated blood I-FABP levels was only detectable 2 h after AT, and I-FABP values returned to baseline by the 24-h time point [21]. However, several mechanisms may contribute to the posttraumatic upregulation of CDH1 due to its multiple roles beside barrier function such as cell renewal and correlation with early barrier restoration [34]. In addition, CDH1 is essential for the maturation of goblet and Paneth cells, reinforcing mucosal defense mechanisms [35]. Consistent with these functions, murine treatment with CDH1-activating monoclonal antibodies attenuated inflammation and increased markers of barrier integrity [36]. Moreover, cytokine-stimulated IECs exhibit increased CDH1 expression levels [37], possibly contributing to the observed changes.

Evidence of such counter-regulatory barrier-reinforcing responses was also seen at the mucus level. Histological analysis revealed a reduction in mucus-covered villus surface and a lower number of actively secreting goblet cells after trauma, despite an unchanged total goblet cell count. These findings suggest a transient depletion of preformed mucus reserves rather than a loss of goblet cells. The intravillous reduction in mucus stores may be attributed to the trauma-induced propulsion of additional mucus beyond basal secretion. This response may serve to prevent bacterial translocation across the epithelial cell layer and infiltration into intestinal crypts [38]. This observation aligns with the reduced number of mucus-secreting goblet cells detected in traumatized mice, as the enhanced luminal propulsion occurs within minutes post-injury and may already be depleted at 24 h post-trauma. Following mucus secretion, goblet cells likely undergo a phase of cellular repair, subsequently leading to the replenishment of their mucin granule stores [38].

At the molecular level, the significant upregulation of *Muc2* and *C1galt1*, genes encoding the major mucin component and its glycosylation enzyme, supports active replenishment processes following post-traumatic secretion. MUC2 expression is predominantly regulated by the transcription of the *Muc2* gene and modifications within the promoter region [39]. Additionally, C1GALT1 plays a crucial role as an essential enzyme required for mucin-type O-glycosylation, contributing to the structural integrity of the mucus layer [40]. In human intestinal organoids, mucin production and secretion, including MUC2, reached peak levels within 48 h after exposure to bacteria. O-linked glycosylation of mucins was highly enriched by bacterial contact [40], further supporting our findings and indicating specific regulatory mechanisms. In summary, the elevated transcriptional activity likely reflects an early attempt to restore mucus coverage, which may not yet be histologically detectable at 24 h post-trauma.

Gene expression analysis of *Il6* in the jejunal tissue was not altered 24 h after blunt AT. Therefore, other inflammatory modulators and cytokines seem to be involved in the post-traumatic inflammation as detected by proteome profiling. Furthermore, it should be considered that the extent of the trauma might not be sufficient to sustain elevated *Il6* expression at 24 h post-trauma, particularly in cases of mild or rapidly repaired injury, as the magnitude of *Il6* upregulation is closely correlated with the severity of tissue damage [41], which was not observed to a significant extent in the present study. Moreover, in our previously published study employing the same model of blunt AT, but with increased injury severity, plasma IL-6 concentrations had already returned to baseline levels 6 h after trauma [20]. This is consistent with unaltered C5a plasma levels 24 h following traumatic exposure, suggesting that posttraumatic systemic inflammation has already subsided by this time. In contrast, local inflammatory activity appeared to persist. Upregulated *S100a8* together with increased CRAMP suggests activation of S100A8/A9-associated antimicrobial responses in the jejunum – also supported by cytokine analysis – although the exact cellular source remains unclear. Despite unchanged neutrophil numbers, elevated *S100a8* expression may reflect enhanced neutrophil functional activity. The absence of neutrophil accumulation in the jejunum may relate to competing inflammatory sites, such as the lung, where remote injury is known to trigger neutrophil influx [42, 43] and where we observed local inflammation, evidenced by increased C5a. Moreover, S100A8 can indirectly promote CD3^+^ lymphocyte homing via activation of VCAM-1 on endothelial cells, thereby facilitating T cell adhesion and migration into jejunal tissue [44], where a posttraumatic influx was noted. The jejunum possesses a complex local innate defense system, primarily mediated by epithelial cells, Paneth cells, macrophages, and dendritic cells, which together provide substantial baseline protection [45]. Neutrophils are naturally scarce within jejunal villi and typically infiltrate this compartment only during pronounced acute inflammation or infection. Their migration is largely confined to submucosal vessels, and AT alone may not cause sufficient chemotaxis to drive villous recruitment, thus not altering posttraumatic counts [46]. In contrast, lymphocytes constitute a substantial resident population in the villous epithelium [47]. Thus, the post‑traumatic immune response in the jejunum appears to favor an increase and possibly activation of adaptive lymphocyte populations rather than an expansion of neutrophil‑mediated innate defense. Taken together, these results suggest a subsided systemic inflammatory response alongside sustained organ-specific (adaptive) immune responses.

Finally, several limitations of this study should be acknowledged. In accordance with the principles of the 3Rs (Replacement, Reduction, and Refinement), the number of animals per experimental group was low, which inevitably limited the statistical power of the findings. Moreover, the exclusive use of male mice represents a further constraint, although it is consistent with the epidemiology of physical trauma affecting predominantly males. However, it is well established that sex-based differences significantly influence trauma outcomes [48] and that estrogen exerts protective effects on intestinal barrier function [49, 50]. Therefore, future murine AT studies should include both sexes to ensure broader biological relevance and translational value. Furthermore, we only assessed one time point following trauma; the 24-hour observation period represents a subacute post-trauma phase and may not reflect delayed pathophysiological changes. Finally, although the model induces intestinal alterations such as macroscopical organ damage and closely reflects the injury pattern of human blunt AT [20], no microscopic injury in histological analyses or molecular damage were detectable. Therefore, the mode of traumatic impact may be further refined in future studies; also, extending the observation period to assess longterm systemic and intestinal consequences of AT would be particularly valuable. Such investigations could provide important insights into the regeneration of epithelial barrier function, posttraumatic alterations of the gut microbiome, systemic bacteremia, local and systemic inflammatory responses, and, importantly, the contribution of the intestine to the development of MODS.

## Conclusion

This study indicates that the intestine mounts a multifaceted protective response following trauma, involving barrier reinforcement, mucus replenishment, and adaptive immune activation. Interestingly, the blunt trauma model employed proved sufficiently intense to elicit a pronounced inflammatory response, as well as an altered posttraumatic mucus production. However, contrary to most prevailing trauma reports in the literature, this did not result in a loss of barrier proteins. This finding raises the possibility that mild trauma may trigger a compensatory upregulation or mechanisms that counteract a loss of barrier components. Given the potential for severe, life-threatening consequences of intestinal barrier dysfunction, rigorous research is imperative to further elucidate underlying mechanisms and optimize therapeutic interventions, thereby improving post-traumatic patient outcomes.

## Electronic supplementary material

Below is the link to the electronic supplementary material.

Supplementary Material 1

## Data Availability

All data are contained within the manuscript or its supporting files.
